# Bladder irrigation and urothelium disruption: a reminder apropos of a case of fatal fluid absorption

**DOI:** 10.1186/1471-2490-14-91

**Published:** 2014-11-20

**Authors:** Marco Di Paolo, Valentina Bugelli, Alessandro Di Luca, Emanuela Turillazzi

**Affiliations:** Section of Legal Medicine, Santa Chiara Hospital, University of Pisa, via Roma 55, Pisa, 56126 Italy; Department of Anatomical, Histological, Forensic and Orthopaedic Sciences, University of Rome Sapienza, Viale Regina Elena 336, 00161 Rome, Italy; Department of Forensic Pathology, University of Foggia, Ospedale Colonnello D’Avanzo, Viale degli Aviatori 1, Foggia, 71100 Italy

**Keywords:** Bladder irrigation, Fluid absorption, Pulmonary edema, Urothelium disruption

## Abstract

**Background:**

Irrigation or washouts of the bladder are usually performed in various clinical settings. In the 1980s Elliot and colleagues argued that urothelial damage could occur after washouts and irrigations of the bladder. The exact mechanism underlying urothelial damage has not yet been discovered. To our knowledge, this is the first report of fatal fluid overload and pulmonary edema, due to urothelium disruption occurring during bladder irrigation, approached performing complete histological and immunohistochemical investigation on bladder specimens. The exposed case deserves attention since it demonstrates that, although very rarely, irrigation or washouts of the bladder may have unexpected serious clinical consequences.

**Case presentation:**

An 85 year-old Caucasian man, unable to eat independently and whose fluid intake was controlled, underwent continuous bladder irrigation with a 3-way catheter due to a severe episode of macrohematuria. During the third day of hospitalization, while still undergoing bladder irrigation, he suddenly experienced extreme shortness of breath, breathing difficulties, and cough with frothy sputum. His attending nurse immediately noted that there was no return of the fluid (5 liters) introduced through bladder irrigation. He was treated urgently with hemodialysis. At the beginning of the dialysis treatment, the patient had gained 7.4 kg since the previous measurement (24 hours prior) without any clear explanation. Although a significant weight loss (from 81 to 76 kg) due to the dialysis procedure, the patient died shortly after the final treatment. The autopsy revealed that the brain and the lungs were heavily edematous. Microscopic examination of bladder specimens revealed interstitial and mucosal swelling, and loss of the superficial cell layer. Intermediate and basal urothelial cells were preserved. Altogether the abovementioned findings were suggestive of a diffuse disruption of the urothelium. In conclusion the death of the man was attributed to an acute severe pulmonary edema due to massive fluid absorption.

**Conclusion:**

Our case demonstrates that urothelium disruption may occur during irrigation and washouts of the bladder, also in the absence of other well-known predisposing conditions. Inappropriate use of bladder irrigation should be avoided and a close attention is required of the fluid balance is mandatory when irrigating the bladder.

## Background

The urinary bladder has certain unique anatomical and histological features and works as an effective barrier between blood and urine, mostly due to the urothelium layer which covers the bladder’s surface and is known as the bladder permeability barrier [1, 2]. Under normal conditions, a passive diffusion of solutes and water can occur through the bladder walls, regulated by the tight cellular junctions of the umbrella cells and the high electrochemical gradient existing between blood and urine [1, 2]. Low diffusive water permeability is one of the main features of a physiologic bladder [3]. In certain circumstances, the urothelium can lose its function, allowing the reabsorption of fluid and solutes into the bloodstream. The barrier function can be disrupted in the event of bacterial infection (bacterial cystitis), toxic chemicals, radiations, or during a nonbacterial, nonchemical inflammatory response. All these causes can lead to the loss of the urothelial barrier function, either by a direct effect on the urothelial cells or as a secondary effect of inflammation [3]. Mechanical damage is known to alter the protective urothelium function [3]. Irrigation and washouts of the bladder have been correlated with the disruption of the urothelium [4].

With this paper we aim to confirm the potential effect of bladder irrigation on the urothelium by means of a complete histological and immunohistochemical study on bladder specimens in an 85 year-old Caucasian man, who underwent bladder irrigation and who suddenly developed severe fluid overload and died due to a refractory pulmonary edema.

## Case presentation

An 85 year-old Caucasian man, affected by chronic renal failure, had been undergoing hemodialysis treatments three times a week for the previous three years. His fluid intake was controlled and he was unable to eat independently. Due to a severe episode of macrohematuria (International Normalized Ratio, I.N.R. 6.1), he was transferred to the local hospital where continuous bladder irrigation was promptly activated with a 3-way catheter. Irrigation fluid with 0.9% saline solution was injected from a height of about 150 cm without pressure. A urine bacteriologic culture test was immediately performed, whose results, obtained some days later, were negative. During the first three days of irrigation, the patient did not claim any problems and successfully underwent hemodialysis as usual. On the third day of his hospital stay, while still undergoing bladder irrigation, he suddenly experienced extreme shortness of breath, breathing difficulties, and a cough with frothy sputum. The nurse attending him immediately noted that there was no return of the fluid (5 liters) introduced through bladder irrigation. No manual evacuation of the bladder was performed during the treatment. A chest radiography revealed cardiomegaly, mild perihilar interstitial infiltrates and an increase of the pulmonary vasculature leading to pulmonary edema. An electrocardiography revealed nonspecific ST-T wave changes. Cardiac isoenzymes were within normal ranges; hemoglobin values were constantly >7 g/dL. A computed tomography (CT) of the abdomen was immediately performed and excluded the perforation of the bladder walls and an intraperitoneal fluid extravasation. The patient was treated urgently once again with hemodialysis. At the beginning of the dialysis, the patient had gained weight for 7.4 kg since the previous measurement (24 hours prior) without any clear explanation. Despite a significant weight loss (from 81 to 76 kg) following the dialysis, the patient died shortly after the final treatment.

**Figure 1:**
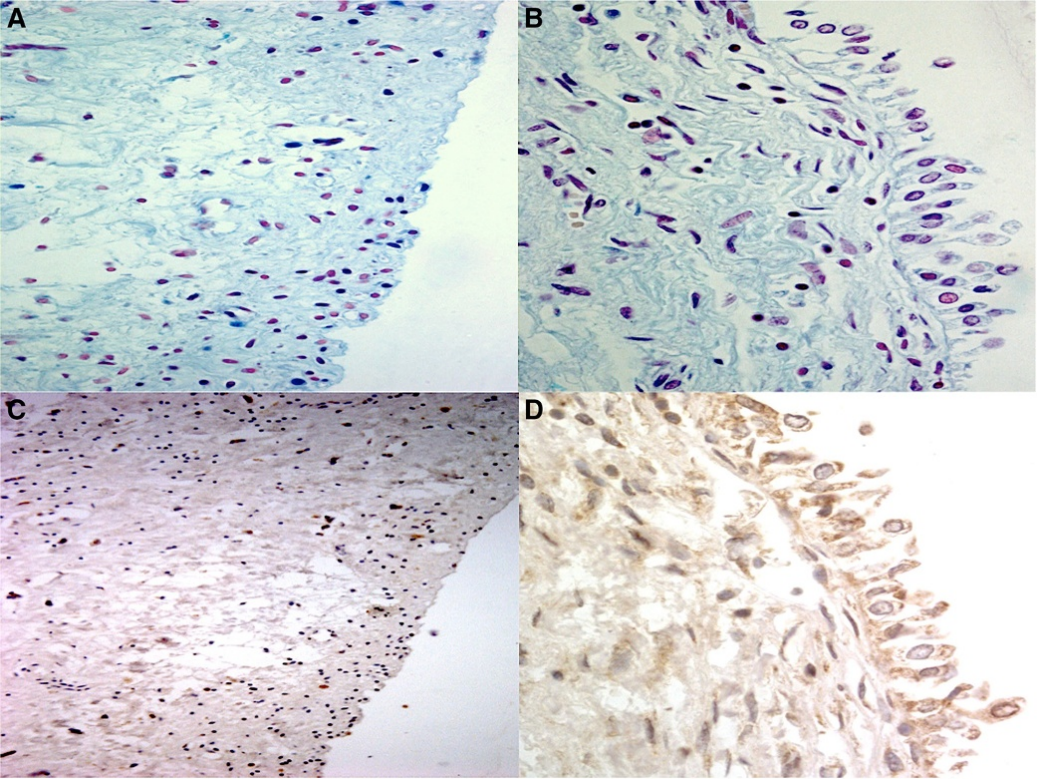
**Alcian Blue - PAS stained sections of (A) damaged bladder case (60 × magnification) and (B) control bladder (100× magnification).** In A umbrella cells are completely denuded. AE1/AE3 immunoreaction confirms the superficial layer damage in **C** (60 x) if compared with the control case (**D**, 100x).

The prosecutor opened an investigation, ordering that an autopsy was to be performed to clarify the exact mechanism of death. The autopsy was executed 24 hours after the patient's death. The results revealed that the brain and the lungs were heavily edematous: the brain weighed 1700 g, the left lung weighed 790 g while the right lung weighed 590 g. The heart was found increased in size (12 × 8.5 × 8 cm) and weight (660 g), coronary vessels and main branches showed slight stenoses. The kidneys were small and showed a finely granular external surface; in cross-section, a gross diminution of cortical thickness with loss of demarcation between cortex and medulla was evident; each kidney weighed 100 g. A small amount of soft blood clots was found in the bladder. Other organs were unremarkable. Samples of organs were taken for histological examination and sections were stained by Hematoxylin & Eosin. On bladder specimens an immunohistochemical study using mouse monoclonal anti-pan cytokeratin antibody AE1/AE3 was performed in order to investigate the urothelium cells. A microscopic examination of the bladder specimens revealed interstitial and mucosal swelling; loss of the umbrella cell layer (superficial urothelium) was evident while intermediate and basal urothelial cells were preserved. The bladder section expressed a very low positivity to AE1/AE3 antibody in comparison with a control case (bladder specimens in a 70 year-old man who had died of pulmonary embolism) which revealed a positive labeling with both AE/1 AE/3 antibodies. PAS and Alcian blue staining demonstrated a clear absence of the superficial layer of glycosaminoglycans (GAGs) in comparison with the control case (Figure 1). Altogether the abovementioned findings were suggestive of a diffuse disruption of the urothelium.

The histological examination of other organs was unremarkable except for a massive cerebral and pulmonary endoalveolar edema, myocardial colliquative myocitolysis and mild diffuse arteriolosclerosis.

In conclusion the death of the man was attributed to an acute severe pulmonary edema due to massive fluid absorption.

## Discussion

In the 1980s, Elliot and colleagues argued that urothelial damage could occur after washouts and irrigations of the bladder. The authors measured the rate of exfoliation of urothelial cells following bladder washouts, and detected an increase in the shedding of urothelial cells in patients with long-term indwelling catheters and chronic urinary tract infections, thus suggesting that the bladder irrigation further damages an already disrupted urothelium. The exact mechanism leading to the urothelial damage was unclear but the author's suggestion was that the phenomenon could be related to the mechanical forces of instillation and the effect of antiseptics. Further investigation by the same group outlined the role of the physical force of irrigation when physiological saline solutions were involved in the therapy [4–6].

It is well known that bladder wall damage can be directly caused by chemical solutions, excessive heat, radiation, calculi, infection, prostatic electrocoagulation and carcinoma [3].

In particular, in the treatment of kidney stones through chemical dissolution many chemical solutions, either Ph dependent or chelating agents, can damage the urothelial tissue [7]. In a rabbit model, Zhang et al demonstrated that calcium dissolving agents can cause histological alterations of the bladder mucosa that include edema, urothelial cell damage and neutrophil infiltration [8].

In the present case, the patient did not experience any bladder trauma; urinary infections were excluded through a urine bacteriologic culture test. In addition, the postmortem examination showed no signs of bladder wall perforation, bladder tumors nor other macroscopic alterations of the bladder mucosa. Irrigation was performed through a traditional gravity system with a normal saline solution; intraperitoneal fluid extravasation was also excluded by an abdominal CT. The histological and immunohistochemical study of bladder samples revealed a highly damaged urothelium, and mucosal and interstitial swelling.

## Conclusions

We believe that the irrigation of the bladder induced the urothelium disruption leading to a massive fluid absorption (approximately 5 liters), responsible for the serious hemodynamic effects observed.

This clinical case suggests that severe symptoms, due to fluid absorption, may also manifest themselves following irrigation of the bladder with a normal saline solution, not unlike many other endoscopic surgical procedures requiring the use of irrigating fluids used to dilate the operating field or to wash away blood and debris [9]; it also confirms that the urothelium disruption may occur during irrigation and washouts of the bladder even in the absence of other well-known predisposing conditions, such as chemical solutions, excessive heat, radiation, calculi, infection, prostatic electrocoagulation, and carcinoma.

The exposed case deserves attention since it demonstrates that, although very rarely, irrigation or washouts of the bladder may have unexpected serious clinical consequences. Inappropriate use of bladder irrigation should be avoided and a close attention is required regarding the fluid balance when irrigating the bladder. Although in the absence of specific contraindications to bladder irrigations, patients with coexisting congestive heart failure and chronic renal failure have an increased risk of complications as the one we reported. A closer monitoring should be appropriate in these patients since they may not tolerate any increase in preload and would be more likely to experience pulmonary edema.

## Consent

Written consent was obtained from the son of the deceased.

## Authors’ information

MDP: Researcher Section of Legal Medicine, University of Pisa. VB: Trainees, Section of Legal Medicine, University of Pisa. ADL: Trainees, Department of Anatomical, Histological, Forensic and Orthopaedic Sciences, University of Rome Sapienza, Rome, Italy. ET: Full Professor and Chairman. Department of Forensic Pathology, University of Foggia, Italy.

## Abbreviations

CT: Computed tomography
GAGs: Glycosaminoglycans
I.N.R: International normalized ratio.
